# Body and organ weight data in 28-day toxicological studies in two mouse strains

**DOI:** 10.1016/j.dib.2019.104632

**Published:** 2019-10-07

**Authors:** Heike Antje Marxfeld, Karin Küttler, Martina Dammann, Sibylle Gröters, Bennard van Ravenzwaay

**Affiliations:** Experimental Toxicology and Ecotoxicology, BASF SE, 67056 Ludwigshafen, Germany

**Keywords:** Organ weight, Mouse, Immune system, Toxicity, Endocrine system

## Abstract

Toxicological studies were performed in an AAALAC (American Association for Laboratory Animal Care)-approved laboratory at BASF SE, Ludwigshafen, Germany, in accordance with the German Animal Welfare Act and the effective European Council Directive 2010/63/EU. Data were recorded in the BASF SE pathology data capture system.

Historical control data (2008–2013) were compiled for

a) Twelve 28-day studies performed according to OECD 407 with mice from Janvier C57BL/j Rj (J) and Charles River CD-1 (CRL), in total 73 control females and 73 control males, 5–8 weeks old at the beginning of the studies. Data collected: terminal body weight, organ weights of adrenal glands, brain, epididymides, heart, kidneys, liver, ovaries, prostate, seminal vesicles (with coagulating glands), spleen, testes, thymus, uterus.

b) Eight immunotoxicity studies (duration of 28 days) performed according to EPA Health Effects Test Guidelines OPPTS 870.7800 with mice from Janvier C57BL/j Rj (J). 48 control females and 16 control males 5–7 weeks old at the beginning of the studies. Data collected: terminal body weight, mean absolute and relative weights of spleen and thymus.

This data helps interpreting effects caused by treatment in toxicology studies in the mouse.

Coefficients of variation were calculated and discussed in the accompanying research paper:

“Variance of body and organ weights in 28-day studies in mice” (Marxfeld et al. 2019).

**Table:** Specifications Table

Subject	Toxicology
Specific subject area	Organ weight measurement and interpretation in toxicological studies in the mouse
Type of data	Table
How data were acquired	Body and organ weights recorded and transferred to proprietary in-house data capture system Balances: Mettler Delta Range XS 603 S, PM 480, Toledo XS6002S, AE 160
Data format	Raw
Parameters for data collection	Body and organ weights of mouse studies performed from 2008 to 2013 at BASF SE, Ludwigshafen, Germany
Description of data collection	Weighing of organs and carcass during routine necropsies performed according to OECD guideline 407 [1] and EPA Health Effects Test Guidelines OPPTS 870.7800 [2]
Data source location	Institution: BASF SE City/Town/Region: Ludwigshafen Country: Germany
Data accessibility	With the article
Related research article	[3]

**Table:** 

**Value of the Data** • Why are these data useful? Show variability of organ weights in the mouse, help interpreting effects caused by treatment in toxicology studies if control variability is known • Who can benefit from these data? Everyone conducting mouse studies where weights are taken not only in a regulatory context • How can these data be used for further insights and development of experiments? Data provided can be reanalysed, compared to other data sets in other mouse strains, rats etc • What is the additional value of these data? So far no data set of this kind is available as far as we know, the guideline OECD 407 [1] asks for justification, if other rodents than rats are used, this data help in assessing the suitability of the mouse for this study type

## 1 Data

The dataset contains organ weights (both absolute and in relation to body weight) of mice with information about the mouse strain and age of animals. Data are provided in excel format containing the following data (Table in Supplementary data):

a) Twelve 28-day studies performed according to OECD 407 with mice from Janvier C57BL/j Rj (J) and Charles River CD-1 (CRL), in total 73 control females and 73 control males, 5–8 weeks old at the beginning of the studies. Data collected: terminal body weight, organ weights of adrenal glands, brain, epididymides, heart, kidneys, liver, ovaries, prostate, seminal vesicles (with coagulating glands), spleen, testes, thymus, uterus.
b) Eight immunotoxicity studies (duration of 28 days) performed according to EPA Health Effects Test Guidelines OPPTS 870.7800 with mice from Janvier C57BL/j Rj (J). 48 control females and 16 control males 5–7 weeks old at the beginning of the studies. Data collected: terminal body weight, mean absolute and relative weights of spleen and thymus.

**Table:** 

Supplier	Strain	Age at beginning of study	Abbreviation	Number of control mice/sex	Number of studies
Janvier	C57BL/j Rj	5–7wks	j57	15	3
Janvier	C57BL/j Rj	7–8wks	j78	23	4
CRL	CD-1	5–7wks	c57	30	4
CRL	CD-1	7–8wks	c78	5	1

## 2 Experimental design, materials, and methods

Weighing of organs and carcass during routine necropsies performed according to OECD guideline 407 [1] and EPA Health Effects Test Guidelines OPPTS 870.7800 [2]. Body and organ weights recorded and transferred to proprietary in-house data capture system, balances used: Mettler Delta Range XS 603 S, PM 480, Toledo XS6002S, AE 160. No filtering or analyses was done on the data presented here.
